# Modern Theoretical Approaches to Modeling the Excited-State Intramolecular Proton Transfer: An Overview

**DOI:** 10.3390/molecules26175140

**Published:** 2021-08-25

**Authors:** Joanna Jankowska, Andrzej L. Sobolewski

**Affiliations:** 1 Faculty of Chemistry, University of Warsaw, 02-093 Warsaw, Poland; 2 Institute of Physics, Polish Academy of Sciences, 02-668 Warsaw, Poland; sobola@ifpan.edu.pl

**Keywords:** excited-state intramolecular proton transfer, photochemistry, photobiology, quantum chemistry, molecular dynamics, ultrafast processes

## Abstract

The excited-state intramolecular proton transfer (ESIPT) phenomenon is nowadays widely acknowledged to play a crucial role in many photobiological and photochemical processes. It is an extremely fast transformation, often taking place at sub-100 fs timescales. While its experimental characterization can be highly challenging, a rich manifold of theoretical approaches at different levels is nowadays available to support and guide experimental investigations. In this perspective, we summarize the state-of-the-art quantum-chemical methods, as well as molecular- and quantum-dynamics tools successfully applied in ESIPT process studies, focusing on a critical comparison of their specific properties.

## 1. Introduction

Photochemistry of organic molecular systems is an extremely rich and exciting field of research, continuously growing and, thus, pushing forward the frontiers of our understanding of light–matter interactions. Among many chemical processes induced with photon absorption, the excited-state intramolecular proton transfer (ESIPT) stands out with its ultrashort timescale and strong impact on the molecular electronic structure, which is often manifested with large emission Stokes shifts. Relying on a proton exchange between two electronegative centers along a pre-existing intramolecular hydrogen bond, the ESIPT process is recognized to provide a mechanism of excellent photostability to natural and artificial molecular systems [1,2,3], finds applications in fluorescent probes and imaging agents [4,5,6], governs characteristic emission of the green fluorescent protein and its analogs [7,8,9], and opens rich possibilities for multicolor emission in organic light-emitting diodes (OLEDs) [10,11,12,13,14]. Last but not least, ESIPT may also activate other excited-state reaction channels, facilitating the design of complex molecular photo-devices [15,16,17,18,19].

**Figure 1:**
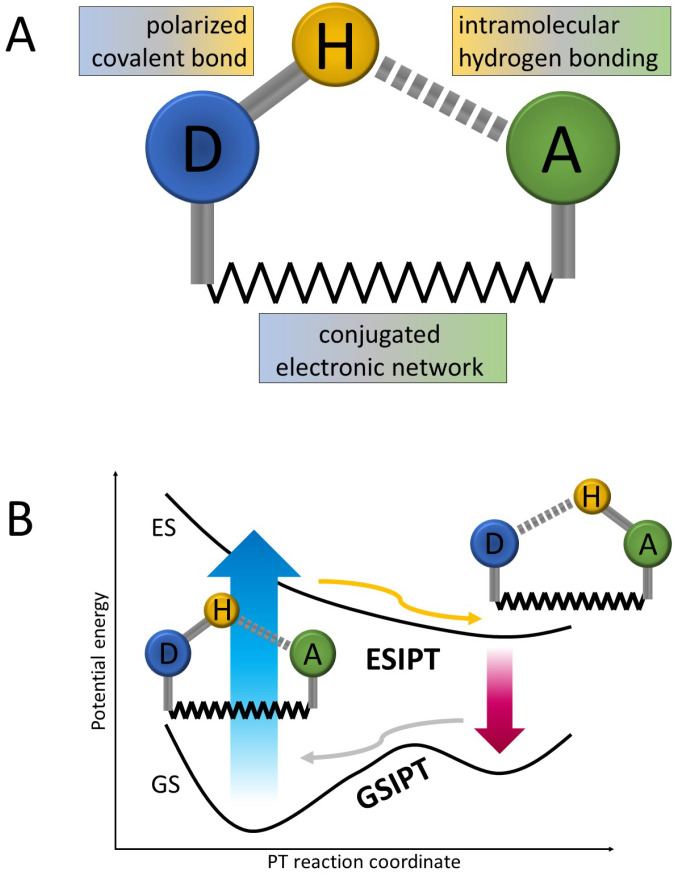
Schematic representation of the ESIPT mechanism: (**A**) initial atomic arrangement of an ESIPT system; (**B**) typical (most basic) potential energy landscape along the ESIPT reaction coordinate. D—proton donor; A—proton acceptor; GS—ground electronic state; ES—excited electronic state; blue arrow—initial photoabsorption; red arrow—Stokes-shifted fluorescence.

In its typical arrangement, ESIPT occurs upon photoexcitation of a molecular system including two moieties connected on the one side by an intramolecular hydrogen bond and with an electronically conjugated network of covalent bonds on the other side, as shown in Figure 1. The reaction occurs in an excited electronic state and is usually being parameterized by the distance between the proton-donor (D) atom (most commonly oxygen or nitrogen [15,20,21]) and the transferring proton. The proton-accepting (A) moiety consists of another electronegative center, often including a carbonyl or an imine group [11,17], which, in order for the ESIPT process to be efficient, should exhibit stronger basicity in the excited state than the proton donor. After the proton transfer, the system undergoes further electronic relaxation—either radiative or nonradiative in nature. In the former case, the characteristic strongly red-shifted fluorescence is nowadays regarded as the hallmark of ESIPT. The latter scenario requires the presence of an independent nonradiative deactivation channel, induced, for instance, by a *cis/trans* isomerization reaction [22]. While the ultrafast ESIPT process is often reported to have ballistic nature (that is, barrierless excited-state potential-energy (PE) landscape in Figure 1), it may also involve passage through an energy barrier or include nonadiabatic transition/intersystem crossing between different electronic states. Similarly, after the relaxation to the ground electronic state, the system may reach a local PT minimum or may undergo a spontaneous back-transfer to the initial D–H bonded isomer. This final reaction-cycle closing transformation is sometimes referred to as a ground-state intramolecular proton transfer (GSIPT).

In the context of the following discussion, it is also important to underline a distinction between the ESIPT reaction investigated herein and similar processes, especially the proton-coupled electron transfer (PCET) reaction [23,24,25,26]. The latter phenomenon, often of nonadiabatic character, has a generally much more complex nature and may involve ground and excited-state reactions, such as intra- and intermolecular, concerted, and stepwise processes. Under certain conditions, ESIPT may play the role of an elementary step in a complex PCET reaction.

In this review, we identify and discuss three fundamental families of theoretical approaches to modeling the ESIPT process: (i) the static methods, (ii) the mixed quantum–classical molecular dynamics, and (iii) the quantum dynamics methods. In the following sections, we briefly outline their theoretical assumptions, comment on the scope of their applicability and performance in ESIPT studies, and highlight recent achievements in each field, focusing on the most illustrative results from the last 5 years. For a broader view of ESIPT-focused research, including also experimental insights, the interested reader is referred to other up-to-date reviews [4,6,21,27,28] and monographs [29,30,31,32] that have been published on the subject.

## 2. Static Investigation Approach

### 2.1. Objective of the Static Calculations

The most straightforward approach to the theoretical characterization of a new photochemical system relies on a “static” quantum-chemical investigation, which itself might be a target research strategy or an initial step of a more complex protocol. The static ESIPT investigation is primarily oriented toward providing high-quality absorption and emission optical energies, as well as the topographical description of the investigated system’s PE landscape. It might also be considered a lower-cost computational option for large polyatomic molecules as this protocol, compared to the dynamic ones, usually involves a relatively limited number of demanding energy-gradient calculations and facilitates further savings by allowing calculations under fixed system symmetry, if such is present. Typical outcomes of the static approach are absorption and emission vertical electronic energies [33,34,35,36], upper-bound estimations for possible energy barriers in the ground and electronically excited states [36,37,38], and detailed characterization of these states in terms of symmetry and orbital configuration [38,39]. Moreover, the number of other molecular features complementing the experimental ESIPT characterization can be determined, including, e.g., tautomers relative energies [35,40], atomic charges [41,42,43], and vibrational modes attribution [44,45].

### 2.2. Typical Investigation Workflow

**Figure 2:**
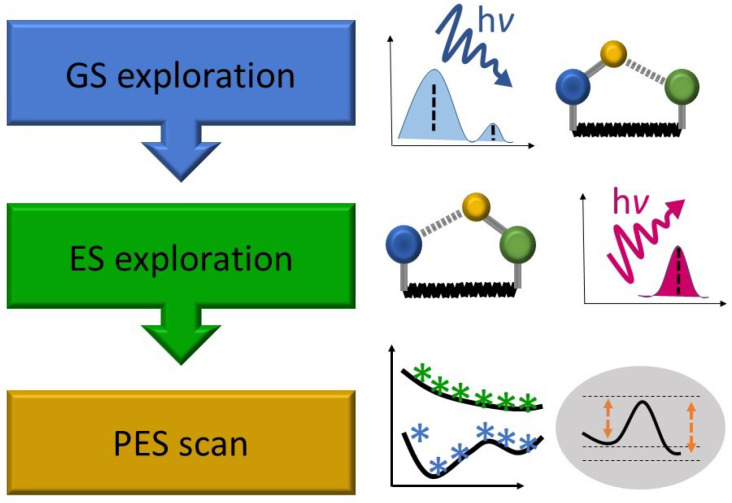
Typical workflow of the static ESIPT investigation protocol.

A typical workflow scheme of the static protocol is presented in Figure 2. In the first step, a search for stationary points on the ground-state (GS) potential energy surface (PES) is performed, followed by vertical electronic excitation energies calculations. At this stage, the electronic structure of the GS and the character of the relevant excited states (ES) need to be carefully evaluated, with a special focus on expected requirements for the excited-state methods to be applied in the following steps. Afterward, an analysis of ES relaxed properties is conducted, and the barrierless/barrier-restricted character of ESIPT is determined by excited-state geometry optimization of the relevant isomers, along with predictions for the energy and intensity of the Stokes-shifted fluorescence [38,46]. In the final step, adiabatic potential energy profiles (PEPs) [47,48] or PESs [3,33] may be calculated, if one or multiple reaction coordinates, respectively, need to be explicitly considered. In certain cases, the energy profiles of linearly interpolated reaction paths might also efficiently support the static ESIPT analysis [49]. However, in accordance with the static protocol name, it should be stressed that neither dynamic nor kinetic effects beyond the zero-point energy (ZPE) corrections to electronic stationary-point energies are included at this level of theory.

### 2.3. Applied Tools and Methods

In principle, the static ESIPT investigation can be performed with any kind of electronic structure method capable of treating the electronic structure of excited states in a relaxed manner. Typically, single-reference electronic structure methods can be trusted to reproduce accurately the topography of the involved electronic states, with the exception of an anti-Kasha ESIPT [50] or systems with low-lying doubly excited states [42,51]. The choice of the optimum electronic structure method for a particular ESIPT study is usually dictated, on the one hand, by the size of the molecular system and, on the other, by its specific electronic structure features. An important general observation is that the correct characterization of the PES topography of the proton-transfer process necessarily requires the inclusion of dynamic electronic correlation effects, as artificial or overestimated reaction barriers have been reported otherwise [52,53,54].

#### 2.3.1. Ab Initio Wave Function Approaches

For the smaller molecular systems (generally, up to 50 heavy atoms), coupled-cluster electronic structure methods, such as the simplified version of singles and doubles, CC2 [55,56], and the algebraic diagrammatic construction method (ADC(2)) [57,58], have been the methods of choice for a long time [3,34,46,49,59]. The reason is their universality [60] and the availability of well-tested and efficient implementations in widely distributed quantum-chemical software packages. While the CC2 method yields overall slightly more accurate electronic excitation energies [58], the virtue of the ADC(2) approach lies in better numerical stability near-electronic excited-state crossings [61,62,63].

At the same time, regarding the recent reports of previously unrecognized troubles of the CC2 and ADC(2) methods in predicting accurate excited-state PES beyond the Franck–Condon vicinity [64,65], spin-component scaled CC2 (SCS-CC2 [66]) and scaled opposite spin CC2 (SOS-CC2 [67,68]) approaches have been found particularly promising in the context of ESIPT studies. In this direction, one of us recently employed both these protocols in combination with the ADC(2) method to model photophysical transformations in several salicylaldimine derivatives [34], observing indeed their improved performance for ESIPT-driven fluorescence energy calculations; similar results have also been reported by Kielesinski et al. for coumarins [69]. In this latter work, performed quantum-chemical investigation yielded correct predictions of solvatochromic effects in a series of compounds, studied both by experimental and theoretical means. Moreover, a direct explanation for single- and multicolor emission observed experimentally in closely related coumarin systems has been formulated on the grounds of a detailed computational analysis of the lowest-energy electronic excited states’ properties.

#### 2.3.2. Density Functional Theory Methods

The second widely applied family of electronic structure methods for ESIPT investigations is time-dependent density functional theory (TD-DFT [70]), in its original design and within the Tamm–Dancoff approximation (TDA-DFT [71]). Abundant ESIPT studies at this level of theory [33,40,46,48,72,73] take advantage of the favorable scaling of DFT with the system size. At the same time, due to known difficulties of TD-DFT with the description of charge-transfer states, and more recent findings on its troubles with the proper determination of state orders in inverted singlet/triplet systems [74,75], the choice of the exchange-correlation functional and method validation usually need to be carefully conducted before meaningful conclusions can be formulated [36,59,76].

In recent years, many functionals of different types have been employed in ESIPT studies [59]. In particular, the popular Becke three-parameter Lee–Yang–Parr (B3LYP) [77,78] functional was found to perform well for systems exhibiting small or no charge-transfer effect in the excited states involved in the ESIPT reaction [3,33,37,72,73,76]. Other recently applied and promising functionals include hybrid meta M06-2X [38,40,46,79], and long-range and dispersion-corrected ωB97X-D [40,80,81]. Among other reported possibilities, the Coulomb-attenuated hybrid functional CAM-B3LYP [82] has also recently gained a relatively trusted position as a tool for ESIPT investigations [36,73,76,81]. At the same time, none of these functional choices appear to be fully universal as of today [59]. As for the TD-DFT relation to TDA-DFT, the latter shows generally higher stability at the interstate crossings, including improved performance in the vicinity of conical intersections [83], even those involving the reference electronic state, and allows for some additional computational-time savings [81,84], appearing particularly attractive in the context of ESIPT dynamics simulations.

Finally, due to known DFT deficiencies in describing dispersion interactions [85], it is worth noting the role of these effects in ESIPT modeling at the TD-DFT level. It is observed that a suitable correction, such as D3 or D4 as proposed by Grimme et al. [86,87] or direct application of a dispersion-corrected functional (e.g., ωB97X-D) is typically required for proper treatment of microsolvated, supramolecular, or condensed-phase (e.g., crystal) systems, in which explicit interactions between the core molecule and the environment have to be included [88,89,90]. On the other hand, in most other cases, the omission of the dispersion part of interaction energy does not seem to play a significant role, as revealed by the generally good performance of common uncorrected exchange-correlation functionals [59,76].

#### 2.3.3. Basis-Set Choice

Practical application of the methods discussed above requires making the additional choice of a basis set for the wave-function expansion, which has a direct impact on the quality of the results. In this case, again one needs to make a compromise between the computational cost and desired accuracy. Most common recent choices in ESIPT studies seem to be favoring the cc-pVTZ [91] basis set from the Dunning family on the one hand [3,33,39,40,48], and different variants of the Pople 6-311 G(d,p) [92] basis set, on the other [36,72,73]. The latter direction finds its support in a general study by Laurent et al. [93], in which the basis-set effect on vertical excitation energy calculations was investigated. On the grounds of reported results, however, it is not easy to make definite ESIPT-targeted recommendations for the basis-set choice since both system and ES-specific effects come into play [93].

#### 2.3.4. Solvent Effects

Finally, a brief discussion of the environmental effects is appropriate, as ESIPT systems are usually investigated in solution or in other complex condensed-phase environments. In particular, the polarity of the surrounding medium has been observed to have a strong impact on the ESIPT reaction efficiency [73,76].

Thus far, several different approaches have been employed to tackle environmental effects on ESIPT, including microsolvation [43,69], the conductor-like screening model (COSMO) [48,94,95], the polarizable continuum model (PCM) [76,96,97], the solvent model density (SMD) method [46,98], and the integral equation formalism version of PCM (IEFPCM) [99,100,101]. The latter approach, particularly popular recently [33,37,73,102], has been applied e.g. by Wang et al. to the BTS system [39] in methylene chloride, yielding very high accuracy predictions for excitation and emission wavelengths, with divergence from the experimental values measured in just a few nm. In addition to the general purpose methods listed above, state-specific PCM treatments of correlated linear response (cLR) [103] and the vertical excitation model within the unrelaxed density approximation (VEM-UD) [104,105] have been successfully applied to study ESIPT by Vérité et al. [40], who pointed out the advantages that these approaches bring for the description of charge-transfer states in ESIPT reactions. Nevertheless, the explicit inclusion of (typically few) solvent molecules is necessary in certain cases, especially for protic solvents and solvents exhibiting proton-accepting properties, since resulting competition between intra- and intermolecular hydrogen bond formation may drastically affect the ESIPT reaction yield [88,90,106].

### 2.4. Summary of the Static ESIPT Investigation Methods

To summarize the section dedicated to the static ESIPT investigation protocol, we again underline its strengths as being a relatively affordable and yet informative approach, designed to provide a fundamental characterization of ESIPT, including the system’s absorption and emission properties, as well as information on the topography of GS and ES PESs over pre-selected reaction coordinates. Due to its inherent compatibility with a great variety of electronic structure methods, this protocol allows researchers to take advantage of new developments in electronic structure theory and, thus, constantly provides opportunities for cutting-edge studies of ESIPT in all types of molecular systems.

At the same time, it should be noted that, under certain circumstances, the investigation of static ESIPT paths may not be sufficient. In particular, systems undergoing multiple PT reactions are typically challenging to be accurately studied with this protocol due to the large computational cost of multi-dimensional PES scans, on the one hand, and the critical role of the sequence of individual processes missed at this level, on the other hand. Another situation, in which special precautions should be taken, is when ESIPT occurs within a dense manifold of electronic states, such as in situations in which a competition between various photochemical transformations is to be expected; in these cases, one may need to explicitly determine the relative efficiency of each channel, which usually requires the inclusion of nuclear-dynamic effects.

## 3. Nonadiabatic Molecular Dynamic Approaches

New opportunities of delving deeper into the course of the ESIPT reaction open when one turns toward dynamic approaches. In the most general view, this refers to a large (and growing) number of methods allowing for real-time simulations of molecular systems’ evolution in terms of their electronic and nuclear structure, beyond the static picture.

The time-dependent Schrödinger equation is the core and starting point for the dynamic methods, yielding two broad families of approaches, differed by the level of the applied approximations. The first family consists of fully quantum dynamic (QD) methods, in which electronic and nuclear degrees of freedom are treated both at the quantum-mechanical level. The second family is built on nonadiabatic mixed quantum–classical (NA-MQC) dynamics methods, in which the nuclei, which are much slower than the electrons, are treated at the classical or semi-classical level, propagating under the Newton equation of motion. The quantum–electronic and classical–nuclear subsystems are in this picture connected by the nonadiabatic coupling, ensuring the self-consistency of the description of the total molecular system. We start our discussion of ESIPT nonadiabatic molecular dynamics studies with the NA-MQC methods, which will be subsequently followed by the analysis of the QD performance, in line with the increasing level of the method exactness.

### 3.1. Mixed Quantum–Classical Dynamic Calculations in ESIPT Studies

**Figure 3:**
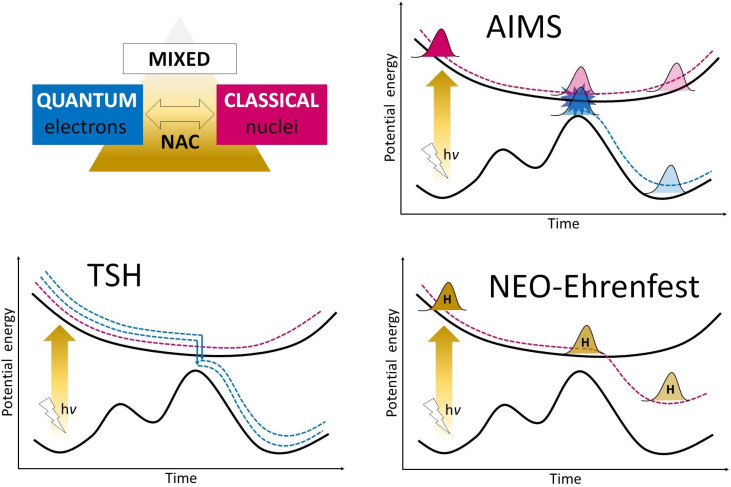
Schematic illustration of the mechanisms of the discussed NA-MQC dynamic approaches: trajectory surface hopping (TSH), ab inito multiple spawning (AIMS), and nuclear–electronic orbital Ehrenfest (NEO-Ehrenfest).

The NA-MQC methods, recently summarized in an excellent review by Crespo-Otero and Barbatti [107], can be divided into several groups, out of which the trajectory surface hopping (TSH) [108,109,110,111], ab initio multiple spawning (AIMS) [112,113], and, most recently, the nuclear–electronic orbital Ehrenfest (NEO-Ehrenfest) [114] methods are, to the best of our knowledge, the ones that have been successfully employed in dynamic ESIPT investigations thus far. Below, a brief characterization of these approaches is provided, along with an illustration of their performance for the description of the ESIPT process. Additionally, a schematic representation of their underlying mechanisms is presented in Figure 3.

#### 3.1.1. Trajectory Surface Hopping Approach

The trajectory surface hopping method, especially under Tully’s fewest-switches (FSSH) [108] algorithm and under other algorithms based on the Landau–Zener (LZ) model [115,116], is the most widely used NA-MQC approach in ESIPT studies thus far. TSH relies on the modeling of the real-time evolution of the molecular system by a set of independent classical trajectories, which together are assumed to represent a nuclear wave packet in an approximate (statistical) way. While the trajectories are propagated on individual Born–Oppenheimer adiabatic PESs, nonadiabatic transitions between these surfaces are possible in regions characterized by large interstate nonadiabatic couplings (NACs). The interstate transitions are controlled by a stochastic algorithm (FSSH) or induced in minimum-energy-gap regions (LZ methods), with the “hopping” probability proportional to the NAC. Importantly, the TSH method can be implemented as an “on-the-fly” approach [117], which means that the actual PES, on which the system is propagating, does not have to be known in advance, and electronic properties, such as energies or gradients, are calculated along the NA-MQC path as needed. It should be noted, however, that usually, many trajectories are required for reliable and converged TSH results [107].

As of today, the TSH method has been implemented in a number of dedicated software packages, including Newton X [118,119], Shark [120], Jade [121], etc. [107]. In all cases, the NA-MQC dynamics protocol has to be paired with an electronic structure method, and its choice needs to be made with great care, as it directly impacts the quality of the results, as well as the simulation cost. One needs to be aware, however, that due to the inherent mixed-classical nature of the TSH approach, certain effects that may play an important role in the ESIPT reaction cannot be reproduced at the TSH level of theory. This includes all phenomena stemming from the nonlocality of the true nuclear wave function, which is reduced to a single point on adiabatic PES within the TSH picture. In particular, proton tunneling, wave-packet interference, and decoherence effects are not included, the latter being partially restored in the TSH simulations via the introduction of various kinds of decoherence corrections [122].

In terms of recent applications of the TSH methodology to particular ESIPT studies, Li et al. reported interesting results explaining 3-hydroxyflavone dual fluorescence in solvents containing protic contamination with a competition between intra- and intermolecular excited-state PT reactions [90], discussing an effect of the number of explicitly included water molecules on the simulation outcomes. In this case, the applied FSSH/TD-DFT methodology allowed for high-quality predictions of the electronic excitation energies (typical error below 0.2 eV) and also yielded ESIPT timescale in very good agreement with available experimental data, with a deviation of less than 10 fs. Another challenging aspect of dealing with a large number of possible photo-reaction products has been tackled by Tuna et al., who employed a robust multiconfiguration interaction variant of the orthogonalization-corrected semi-empirical OM2 approach to model ESIPT-driven photochemistry of urocanic acid [123]. The same method has also been applied by Xia et al. to study relaxation mechanisms in the isolated benzodiazepinone molecule, in which several interconnected relaxation channels come to play [124]. Furthermore, we recently performed a TSH study at the TDA-DFT level to analyze the impact of the character of the lowest excited state on the ESIPT process efficiency [84], eventually confirming the important role of the ππ* states.

#### 3.1.2. Ab Initio Multiple Spawning Approach

Another NA-MQC approach that has found applications in time-resolved ESIPT simulations is the ab initio multiple spawning method. AIMS originates from the formally exact full multiple spawning methodology [125,126]. Its core concept relies on representing the nuclear wave function with partially coupled traveling Gaussian functions, having a finite width both in position and momentum coordinates and interacting during the dynamics. Importantly, the total number of the “on-the-fly” propagated Gaussian functions changes in time since on each passage through a PES region characterized with strong NAC, a new Gaussian is spawned (hence, the S in AIMS).

Similar to the TSH case, AIMS simulations require combining the particular MS protocol with a suitable electronic structure method. As for the AIMS code itself, as of today, it is available within several software packages, including GAMESS [127,128], MOLPRO [129,130], and MOPAC [131,132]. Technically, AIMS involves a higher computational cost than TSH, yet it should be considered a superior approach, inherently including decoherence effects, and yielding a correct description of some non-local phenomena. At the same time, due to certain intrinsic limitations of the AIMS approach, the tunneling effect, although theoretically possible to be covered through the intrastate spawning procedure [112,125], is not reproduced at this level of theory [113].

Turning to recent interesting applications of the AIMS in ESIPT studies, Pijeau et al. investigated the photophysics of the paradigmatic salicylideneaniline (SA) system [133], focusing on the effect of nonplanarity on ESIPT and on the total deactivation mechanism. In this study, the AIMS protocol has been connected with the floating occupation molecular orbital complete active space configuration interaction (FOMO-CASCI) method, with further wave function-in-DFT embedding. The same group also tackled the hydroxyphenyl benzothiazole (HBT) system at this level of theory, obtaining very good agreement with the experimental results [134].

#### 3.1.3. Nuclear–Electronic Orbital Ehrenfest Approach

Recently, a new NA-MQC dynamic approach, NEO-Ehrenfest, aiming toward the further enhanced recovery of nonlocal effects, has been developed. Within this method, protons are treated quantum mechanically on an equal footing with the electrons, yielding automatic inclusion of the ZPE, quantized vibrational levels, and tunneling effects associated with these species [114]. The NEO-Ehrenfest approach, specifically tailored to provide a high-level description of the ESIPT and PCET processes [135], is built on the concept of semi-classical traveling proton basis functions, which, on the one hand, provide means for the quantum-mechanical representation of protons, as has been demonstrated before for the time-independent case [136], and, on the other hand, enable the description of its long-range displacements.

In a recent pioneering NEO-Ehrenfest study by Zhao et al. the ESIPT process in o-hydroxy-benzaldehyde has been investigated [135]. Upon comparison of results obtained using the NEO-Ehrenfest and the traditional Ehrenfest approach [137] with all-classical nuclei, the proton transfer reaction acceleration in the quantum case has been observed, which has been ascribed to the delocalization of the proton wave function, resulting in a smaller necessary displacement of the proton-accepting and proton-donating centers. Moreover, the kinetic isotope effect upon deuterium substitution has been reproduced at this level of theory.

#### 3.1.4. Summary of the NA-MQC Dynamic ESIPT Simulations

To summarize the section dedicated to mixed quantum–classical ESIPT studies, we again highlight the great contributions of the NA-MQD dynamic methods for the field. By allowing real-time picturing of the proton transfer process, characteristic timescales and unforeseen reaction mechanisms can be modeled at this level of theoretical description. While the methods share the mixed quantum–classical nature, they also still bear important differences, making them possible methods of choice for different conditions. In particular, TSH is a robust and probably most universal tool, reliable for the modeling of barrierless ESIPT, including complex situations, in which multiple PTs or competition from other photoreaction channels needs to be taken into account. The AIMS approach, formally more exact, may also be generally applied to this class of processes, as long as it does not become prohibitively expensive due to the extended molecular system size. At the same time, when nuclear quantum effects of protons are expected to play a role, such as in the barrier-restricted ESIPT case, the NEO-Ehrenfest method may be considered a good choice.

### 3.2. Quantum Dynamics Methods for ESIPT Simulations

Despite many useful conclusions on the ESIPT reaction course that may be taken from the NA-MQC dynamics, there are situations in which one needs to advance even further with the level of the system’s dynamic description, up to the point of full quantum treatment of all the species, including nuclei. As has been already pointed out, the most typical reason of adopting this approach is when tunneling through an energy barrier along the ESIPT path needs to be included, i.e., when highly accurate rates or proton-transfer equilibrium have to be characterized. Another situation calling for the QD treatment is when a strongly nonadiabatic ESIPT mechanism is expected, e.g., when trivial interstate crossings are present, potentially threatening the correct NA-MQC dynamics performance. The latter problem, however, has been, in recent years, partially resolved by the successful design of correction strategies to several NA-MQC protocols [138,139,140].

#### Multiconfiguration Time-Dependent Hartree Method

Among the most robust approaches to solving the time-dependent Schrödinger equation that retain the quantum character of all the molecular system’s components, the multiconfiguration time-dependent Hartree (MCTDH) method plays, up to date, the most prominent role [141]. Relying on the Born–Huang expansion, the MCTDH allows for propagating a wave-packet in time with the wave function of the system represented by the sum of products of so-called single-particle functions describing individual nuclear degrees of freedom (DOF), which are typically associated with the molecular normal vibrational modes. The MCTDH method employs model Hamiltonians, constructed individually for each system. In the case of the ESIPT studies, usually, a vibronic Hamiltonian is employed [142,143,144], including a pre-selected number of electronic PESs and nuclear DOFs. It should be noted that MCTDH requires the determination of the PESs prior to the MCTDH calculation. This is typically achieved by combining quantum-chemical probing of the PES regions expected to play the most important role in the investigated process with the application of various interpolation models to approximate the remaining PES areas.

In practical terms, the original MCTDH method can nowadays cover in a general case up to ca. 20 DOFs, but in recent years, new flavors of MCTDH have been developed, such as the multilayer multiconfiguration time-dependent Hartree (ML-MCTDH) method, which pushes this limit even up to several thousand DOFs [145]. The performance boost stems, in this case, from a tree-like (layered) representation of the nuclear wave function, in which the traditional SPFs are further expanded themselves in the MCTDH spirit. The eventual efficiency gain, however, depends strongly on the system’s nature and size [145]. As of today, different variants of the MCTDHF methods are available in a few dedicated software packages, such as the Heidelberg MCTDH [146], or Quantics [147].

Moving to the MCTDHF applications to simulating the ESIPT process, interesting results on the photophysics of hydroxychromones have been reported by Perveaux et al. [148] and Anand et al. [149]. In the former case, the full-dimensional (48 DOFs) ML-MCTDH method was applied to analyze the interplay between the ESIPT reaction and the out-of-plane hydrogen torsion in 3-hydroxychromone, while the latter study comprised analogical simulations for the 3-hydroxychromone and 5-hydroxychromone systems, performed at the multimode MCTDH level with the inclusion of 25 DOFs. Both investigations led to similar conclusions on a critical role of a conical intersection between bright S${}_{1}$ and dark S${}_{2}$ states, of respective ππ* and nπ* character, which was interpreted as the reason for observation of two ESIPT rate constants for these molecules in the experiment. Anand et al. applied the same methodology to study ESIPT also in similar 3-hydroxyflavone [150] and 3-hydroxypyran-4-one [151] systems, confirming the important role of the S${}_{2}$ state in their photorelaxation. Finally, recent thorough work by Cao et al. provided theoretical insights on ESIPT-driven mechanism and quantum dynamics of thermally activated delayed fluorescence in triquinolonobenzene [152], in which singlet-state ultrafast proton transfer occurs within a dense manifold of low-lying triplet states.

## 4. Summary and Future Outlook

In summary, in the present review, we gathered and discussed key features of the modern theoretical approaches employed in ESIPT investigations, with a special focus on their complementary capabilities and critical limitations. Depending on the particular research focus, e.g., manifested by the need for detailed knowledge of ES topography, equilibrium populations of different molecular isomers, or characterization of time-resolved effects, and the system-specific challenges, such as the isolated or band-like arrangement of the active excited states, presence of barrier-restricted or barrierless PT, the necessity of taking the intersystem crossing into account, etc., a proper theoretical approach in each case can be proposed. To this end, we hope that this sometimes challenging choice will be facilitated with the provided insights.

Looking toward future developments that would further strengthen the field, support from machine learning techniques should definitely be considered a promising direction for the QD efficiency enhancement, with first results already emerging [153,154], so as to improve the performance of other dynamic approaches [155]. Moreover, linking solvent-dependent optical properties with nonadiabatic ESIPT dynamics within the fully quantum framework could also provide powerful new tool to the existing set [151], opening new-level possibilities, e.g., for describing the competition between intra- and intermolecular excited-state proton transfer reactions on equal footing.

## Data Availability

All data are available within the article and the included references.
